# RSIADB, a collective resource for genome and transcriptome analyses in *Rhizoctonia solani* AG1 IA

**DOI:** 10.1093/database/baw031

**Published:** 2016-03-28

**Authors:** Lei Chen, Peng Ai, Jinfeng Zhang, Qiming Deng, Shiquan Wang, Shuangcheng Li, Jun Zhu, Ping Li, Aiping Zheng

**Affiliations:** Rice Research Institute of Sichuan Agricultural University, Chengdu, 611130, China; Key laboratory of Sichuan Crop Major Disease, Sichuan Agricultural University, Chengdu, 611130, China; Key Laboratory of Southwest Crop Gene Resource and Genetic Improvement of Ministry of Education, Sichuan Agricultural University, Ya’an, 625014, China

## Abstract

Rice [*Oryza sativa* (L.)] feeds more than half of the world’s population. *Rhizoctonia solani* is a major fungal pathogen of rice causing extreme crop losses in all rice-growing regions of the world. *R. solani* AG1 IA is a major cause of sheath blight in rice. In this study, we constructed a comprehensive and user-friendly web-based database, RSIADB, to analyse its draft genome and transcriptome. The database was built using the genome sequence (10 489 genes) and annotation information for *R. solani* AG1 IA. A total of six RNAseq samples of *R. solani* AG1 IA were also analysed, corresponding to 10, 18, 24, 32, 48 and 72 h after infection of rice leaves.

The RSIADB database enables users to search, browse, and download gene sequences for *R. solani* AG1 IA, and mine the data using BLAST, Sequence Extractor, Browse and Construction Diagram tools that were integrated into the database. RSIADB is an important genomic resource for scientists working with *R. solani* AG1 IA and will assist researchers in analysing the annotated genome and transcriptome of this pathogen. This resource will facilitate studies on gene function, pathogenesis factors and secreted proteins, as well as provide an avenue for comparative analyses of genes expressed during different stages of infection.

**Database URL:** http://genedenovoweb.ticp.net:81/rsia/index.php

## Introduction

Rice sheath blight is one of the three major rice diseases causing severe crop losses (1, 2). The soil-borne pathogen *Rhizoctonia solani* (teleomorph: *Thanatephorus cucumeris*) infects >100 crop and horticulture species. It has been classified into 14 genetically distinct anastomosis groups `(AG1 to AG13 and AGBI), with the *R. solani* AG1 IA subgroup being one of the most important plant pathogen subgroups among the three main *R. solani* AG1 intraspecific groups (ISGs). The ISGs are responsible for multiple diseases, including sheath blight, banded leaf, aerial blight and brown patch (3–5).

As the causal agent of rice sheath blight, *R. solani* AG1 IA appears to be an asexual fungus on rice [*Oryza sativa* (L.)]; however, sexual structures from its teleomorph (*T. cucumeris*) have been occasionally observed in nature. In nature, *R. solani* AG1 IA exists primarily as vegetative mycelium and sclerotia. Furthermore, *R. solani* AG1 IA is the most devastating pathogen in other economically important crops, including corn (*Zea mays*) and soybean (*Glycine max*) (6, 7). Each year, sheath blight causes up to 50% loss in rice yield worldwide under favourable conditions (8, 9), and despite the high rice yield losses caused by *R. solani* AG1 IA, only limited information is available regarding its genetic population structure and mode of reproduction.

Draft genome sequences of *R. solani* AG1 IA strains were generated in 2012 using Illumina GA II technology. Five libraries were constructed, from which the 36.94 Mb draft genome was developed using SOAPdenovo (10). In this study, a comprehensive genomic analysis of *R. solani* AG1 IA was performed. All genes were deposited in the RSIADB database, and analytical tools were integrated for the analysis of *R. solani* AG1 IA. This newly developed database provides the opportunity for users to analyse the *R. solani* AG1 IA genome and detail the genes expressed during different stages of infection.

## Construction and Content of the Database

### System implementation

The server on which RSIADB relies was built using LAMP (Linux Ubuntu Sever 12.04, Apache 2, MySQL Server 5.5 and Perl 5.16.3/PHP 5.3), which is comprised of open source software and provides one of the fastest ways to develop an enterprise-level database. All *R. solani* AG1 IA data were stored in MySQL Tables to facilitate rapid responses from the software. Common Gateway Interface programmes were developed using Perl, JavaScript and PHP programming languages. Scripts for fetching and cutting sequences, searching genes and performing gene expression analyses were also developed. Tools for blasting gene sequences and a drawing diagram were also implemented in the database. The JBrowse Genome Browser was built using HTML5 and JavaScript, and was used to display the positional relationships between genes, scaffolds and genome annotations (11).

### Data and processing

The *R. solani* AG1 IA genome was assembled from 2648 scaffolds. These DNA sequences contained 10 489 open reading frames (ORFs) that were predicted using a combination of annotation methods. Among the ORFs, 4340 were annotated using the UniProt database, while the domains for 5610 ORFs were identified using the Pfam database. A total of 4305 genes were assigned using the EuKaryotic Orthologous Groups database (*E*-value cut-off of 1e^–5^), and 5258 genes with homologues in the NCBI RefSeq fungal protein dataset were assigned using BLASTP (*E*-value cut-off of 1e^−6^). In total, 6156 genes were annotated with 257 genes belonging to the pathogen-host interaction (PHI) database (12). Two ribosomal RNAs and 102 transfer RNAs were also predicted. Meanwhile, This Whole-Genome Shotgun project has been deposited at DDBJ/EMBL/GenBank under the accession code AFRT00000000.

Transcriptome analyses were performed using *R. solani* AG1 IA cDNA libraries that were synthesized from samples of disease lesions after 10, 18, 24, 32, 48 and 72 h infections. Each library was sequenced using a read insert size of 180 bp with Illumina GA II technology, which was deposited in the NCBI SRA under accession number of SRP008735. Transcriptome sequences were mapped to the genome using Tophat (13), and the fragments per kilobase of transcript per million maps read (FPKM) was calculated using Cufflinks (14). Gene expression profiles for the six infection times were then analysed based on the FPKMs. CAZyme genes were identified using BLASTP with an *E*-value of <1e^-5^, and the codes for enzyme classes were defined using the CAZyme database (http://www.cazy.org/) (15).

## Result

### User interface

**Figure 1.**
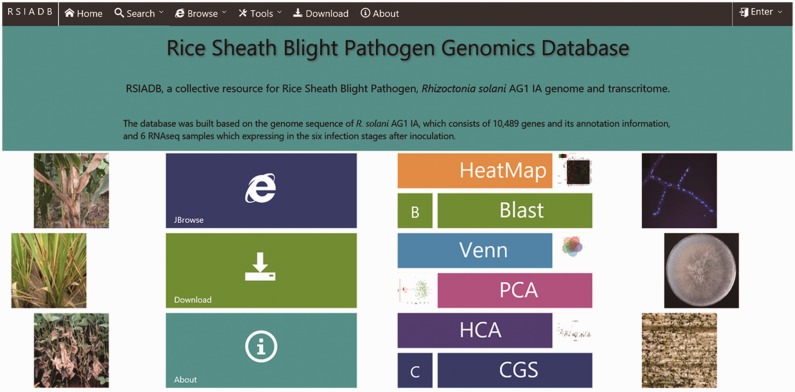
RSIADB organization and description of database functions.

To provide users with an efficient and easy way to access RSIADB data, a clean and user-friendly home page was developed. The user can search and download *R. solani* AG1 IA data and perform analyses (using the analysis tools) using the hyperlinks located on the navigation menu at the top of the page or from a shortcut menu in the middle of the page (Figure 1).

### Search

**Figure 2.**
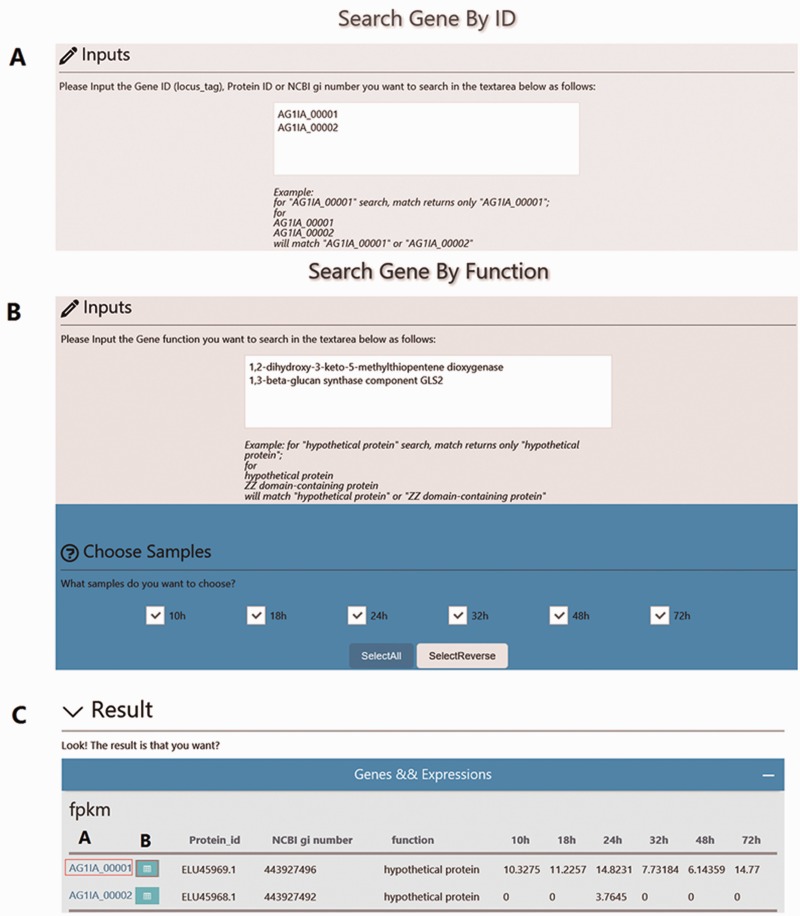
Overview of the ‘Search’ interface. **(A)** Users can search genes by Gene ID (locus_tag), Protein ID or NCBI gi number. For example, Gene IDs ‘AG1IA_00001’ or ‘AG1IA_00002’ can be searched independently; however, multiple Gene IDs are also accommodated. **(B)** Users can also search genes by Gene Function. For example, ‘1,3-beta-glucan synthase component GLS2’ can be searched independently or with additional Gene Functions. **(C)** Search results are provided in tabular format.

**Figure 3.**
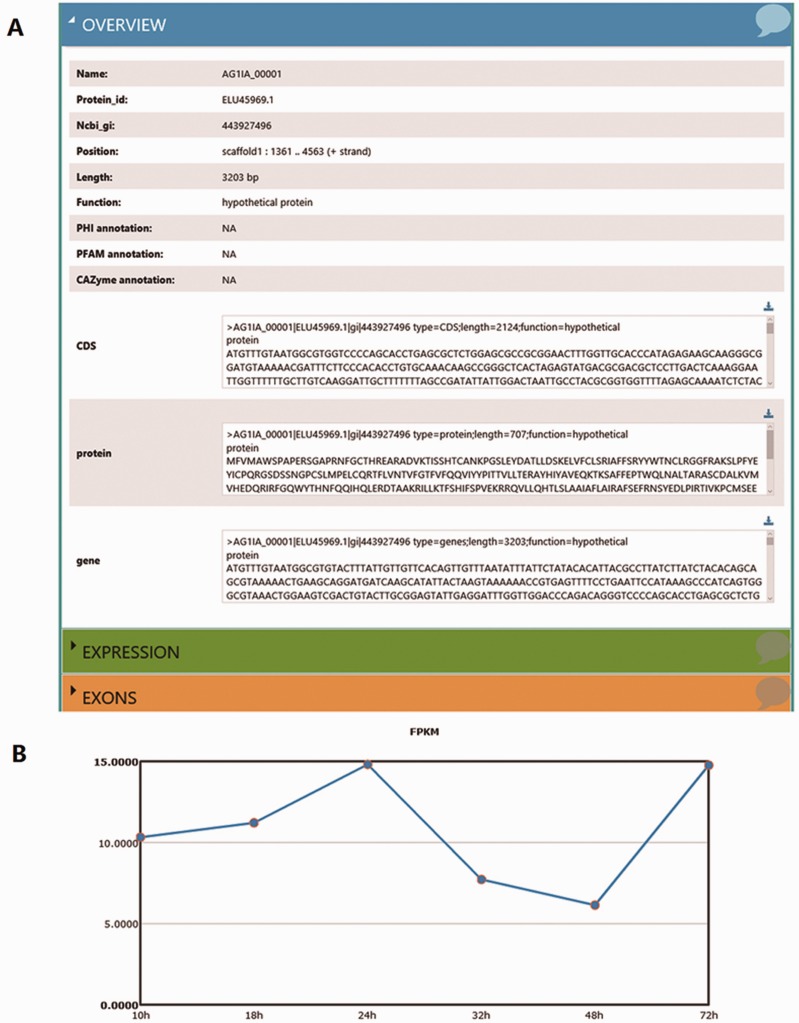
More detailed information derived from the search results. **(A)** GeneBrowse is used to provide detailed information from a search of *R. solani* AG1 IA genes. (**B)** The broken line graph reflects variations in gene expression profiles between infection times.

Using the ‘Search’ function, specific genes in the RSIADB database can be found by their Gene ID or Gene Function. Gene expression information for each stage of infection can also be obtained by selecting the sample of interest (Figure 2). A brief search result can be printed out in tabular format output from the bottom of the search page. In addition, more detailed information is provided by *R. solani* AG1 IA GeneBrowse, and expression profiles at different stages of infection represented by broken line graphs can be obtained by clicking on the red frames ‘A’ and ‘B’ shown in Figure 2C (Figure 3).

### Browse

**Figure 4.**
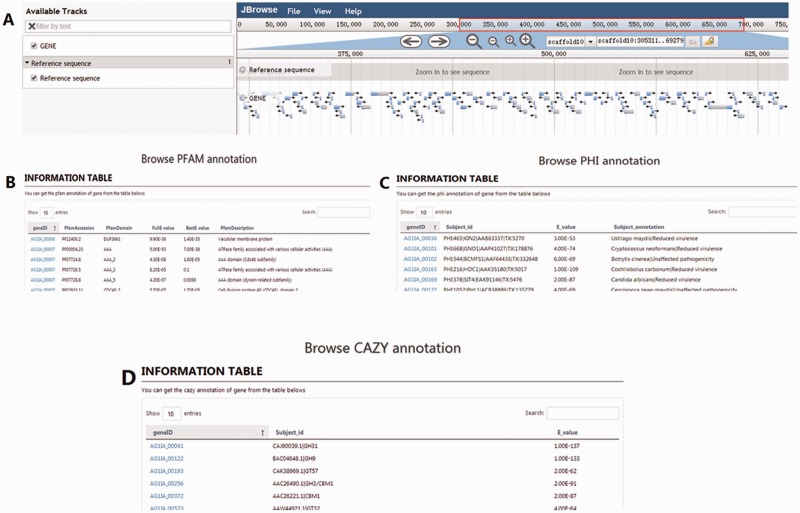
Snapshots of the four tools for browsing data in RSIADB. **(A–D)** show examples of the interfaces for the four browsing methods.

The ‘Browse’ section integrates the analyses of *R. solani* AG1 IA genes, coding sequences, proteins, and transcriptome data. Four menus are provided in this section. In the Genome Browse menu, a large-scale search of *R. solani* AG1 IA data is possible, with the data being represented using a JBrowse graphic interface (Figure 4A). Additionally, the PHI Browse (Figure 4C), PFAM Browse (Figure 4A), and CAZymes Browse (Figure 4D) functions fetch and display detailed and specific gene information using different annotations (Figure 4).

### Tool

**Figure 5.**
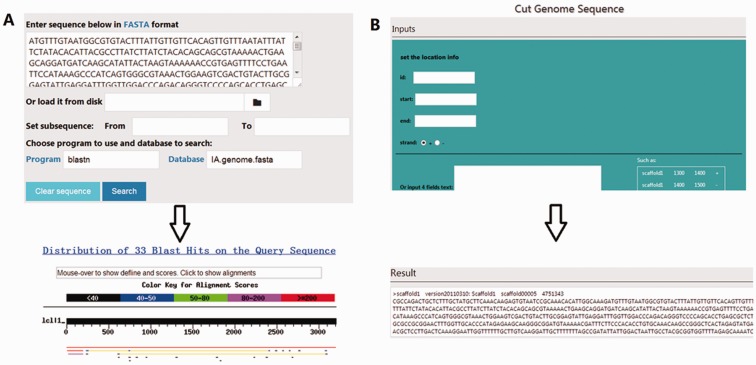
Screenshots of two frequently-used tools in the RSIADB database. **(A)** BLAST interface (top) and BLASTn results (bottom). **(B)** An example of the input and output interface for the Sequence Extractor tool.

**Figure 6.**
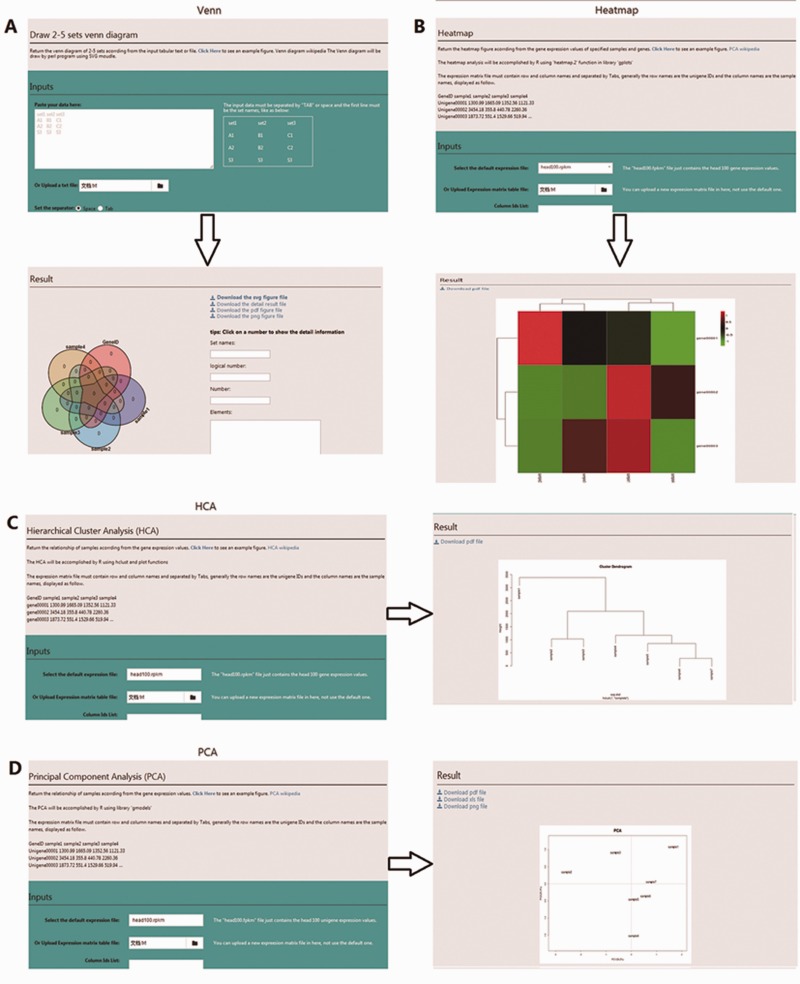
Screenshot of the four user-friendly graphic tools available in the RSIADB database. **(A–D)** show examples of the input and output interfaces for each of the four graphic tools: Venn diagram, HeatMaps, HCA plots and PCA plots, respectively.

To mine, analyse, and visualize the *R. solani* AG1 IA data, three different tools were developed: (i) BLAST, the standard wwwblast model was embedded and allows users to submit query sequences and perform a BLAST homology search against the RSIADB database (Figure 5A); (ii) Sequence Extractor, users can obtain a sequence or sequences by defining a specific genome position (Figure 5B); (iii) Construction Diagram, This tool draws Venn diagrams, HCA plots, PCA plots and HeatMaps to visualize the data (Figure 6).

### Resources

Using the ‘Resources’ section, read data, genome sequences, genes and protein functions can be downloaded using the ‘Download’ hyperlinks under the navigation menu. Users also can download detailed information provided for queries shown in GeneBrowse by selecting the ‘download’ icon.

## Conclusion

The *R. solani* AG1 IA genome sequence and transcriptome data provide a means to detail the gene sequences, gene functions and gene variation tendencies during infection of rice. The RSIADB database provides a user-friendly and comprehensive resource to analyse such data. Using the RSIADB database, researchers can investigate the evolution of *R. solani* AG1 IA and perform comparative analyses between the available Basidiomycete genomes. Moreover, this web-based database provides a means to detail the pathogenic mechanisms causing rice sheath blight.

After release of the RSIADB database, it will be continuously maintained and improved, and will be expanded to include data for *R. solani* AG1 IA, IB and IC. The tools provided in the RSIADB database will also be continuously improved to ensure a user-friendly interface that will facilitate and promote studies on rice sheath blight.

## Availability

Database name: RSIADB (http://genedenovoweb.ticp.net:81/rsia/). All data deposited in the database are freely available to all users without any restrictions.
